# Clinical results of bioresorbable drug-eluting scaffolds in short and long coronary artery lesions using the PSP technique

**DOI:** 10.1186/s12872-018-0994-y

**Published:** 2019-01-18

**Authors:** Christine Reichart, Jochen Wöhrle, Sinisa Markovic, Wolfgang Rottbauer, Julia Seeger

**Affiliations:** 1 Department of Internal Medicine II, University Hospital of Ulm, Ulm, Germany; 2 Head Interventional Cardiology Research Group, University Hospital of Ulm, Albert-Einstein-Allee, 23 89081 Ulm, Germany

**Keywords:** Absorb scaffold, Long lesions, Follow-up, PSP-technique

## Abstract

**Background:**

Data on bioresorbable vascular scaffolds (BVS) for the treatment of long lesions are limited. We studied the use of BVS-Absorb in routine clinical practice and compared the outcome of long lesions with short lesions. Implantation of drug-eluting scaffolds without PSP-technique (predilation, proper sizing and postdilation) is associated with an increased thrombotic risk. We compared the long-term outcome up to 36 months of patients with short (< 20 mm) and long (≥20 mm) coronary artery lesions after implantation of bioresorbable vascular scaffolds (BVS) via PSP-technique.

**Methods:**

Three hundred twenty-six patients with 424 lesions were enrolled in this prospective study and underwent percutaneous coronary intervention with the Absorb BVS. Clinical follow-up was scheduled after 12, 24 and 36 months. In all lesions the PSP-technique was used. The device oriented composite endpoint (DOCE) was defined as cardiac death, myocardial infarction (MI) not clearly related to a non-target vessel and target lesion revascularization (TLR).

**Results:**

Kaplan-Meier estimates for DOCE after 12 months were 2.63% for short lesions and 8.09% for long lesions (*p* = 0.0131), 5.51% vs. 11.35% (*p* = 0.0503) after 24 months and 8.00% vs. 18.00% (*p* = 0.0264) after 36 months of clinical follow-up. Kaplan-Meier estimates for TLR after 12 months were 1.46% for short and 7.69% for long lesions (*p* = 0.0012), 2.06% vs. 8.75% after 24 months (*p* = 0.0027) and 4.96% vs. 9.59% after 36 months of follow-up (*p* = 0.0109). Scaffold thrombosis rates were low.

**Conclusions:**

In long lesions compared to short ones the bioresorbable scaffold Absorb implanted with the proper PSP technique Absorb has significant higher rates of DOCE.

**The Level of Evidence:**

Is 3 (non-random sample).

## Background

Prior studies demonstrated a comparable safety and efficacy for the use of bioresorbable scaffolds Absorb in native and simple coronary lesions with a higher rate of thrombotic events like scaffold thrombosis in multiple randomized trials compared to metallic drug eluting stents [1–3]. These studies showed that without a consequent use of the renewed PSP-technique the scaffold thrombosis rate as well as clinical endpoints were higher compared to metallic drug-eluting stents [1, 4]. The PSP-technique was used in only 8% of lesions in the randomized Absorb III trial leading to a significant higher rate of target lesion failure compared to the everolimus-eluting stent [2]. Previous studies showed that even in very complex lesions like chronic total occlusions the implantation of Absorb BVS with the PSP-technique is associated with a low risk of thrombotic events and good clinical outcomes [5, 6]. From DES-trials it is known that patients with short coronary lesions have lower MACE-rates and a better outcome compared to DES in long lesions [7–10]. In addition, it is well known that BVS with Absorb is associated with a worse outcome compared to DES. Until now data including all comers stenosis with long coronary lesions treated with the bioresorbable scaffolds alone were scarce. Therefore we compared the outcome of long with short lesions treated with the Absorb BVS using the PSP technique with respect to clinical follow-up up to 36 months.

## Methods

We prospectively enrolled 326 patients with 424 lesions treated with an everolimus-eluting bioresorbable scaffold. In accordance to Type C lesions in the ACC/AHA-Score and to the J-CTO scoring system we defined long lesions measuring 20 mm or more in quantitative coronary analysis [11, 12]. A clinical follow-up was performed after 12, 24 and 36 months. Dual antiplatelet therapy was prescribed for 6 months to patients with stable angina and 12 months to patients suffering from an acute coronary syndrome (Table 1). All patients were treated with at least one Absorb BVS (Abbott Vascular, Santa Clara, California, USA). In case of using multiple BVS the scaffold-to-scaffold method was applied as described elsewhere [13]. High-pressure pre-dilation with a non-compliant balloon was mandatory in all treated lesions. For scaffold implantation a slowly balloon inflation with 2 atm every 5 s was used. To finish the process of scaffold implantation with length over 12 mm a high-pressure non-compliant balloon was inflated up to 24 atm in all lesions to achieve a good scaffold expansion with minimized malapposition. The whole implantation process was operated according to the common PSP-technique with predilation, proper sizing and post-dilation [1, 4]. The exclusion criteria were in-stent restenotic lesions and lesions located in the left main coronary artery as well as in large coronary vessels with a reference diameter of more than 4.0 mm. Intravascular ultrasound (IVUS) or optical coherence tomography (OCT) were not routinely used and left to the discretion of the operator. Patients were followed clinically for 12, 24 and 36 months. The device oriented composite endpoint (DOCE) was defined as the primary outcome measure according to Academic Research Consortium (ARC) criteria [14]. This endpoint includes cardiac death, myocardial infarction (MI) not clearly attributable to a non-target vessel and target lesion revascularization (TLR). Written informed consent was obtained from all patients. The study was approved by the ethics committee of the University of Ulm, Ulm, Germany.

**Table 1:** Antiplatelet Therapy

Antiplatelet therapy	Lesion length < 20 mm 190	Lesion length ≥ 20 mm 136
ASS + Clopidogrel	126 (66.3)	79 (58.1)
ASS + Prasugrel	50 (26.3)	38 (27.9)
ASS + Ticagrelor	13 (6.8)	16 (11.8)
ASS + OAK	1 (0.5)	1 (0.7)
Clopidogrel + OAK	0	2 (1.5)

*OAK* oral anticoagulation, data are presented as number of patients (percentage of patients)

### Quantitative coronary angiography

Coronary arteries were quantitatively analyzed before and after device implantation with two orthogonal views. Pre and post PCI minimal lumen diameter (MLF) and reference diameter (RD) were measured. Diameter stenosis and acute gain were calculated. Acute gain was defined as MLD after PCI minus MLD before intervention. For all measurements CAAS Workstation 5.1 (Pie Medical Imaging, Maastricht, The Netherlands) was used.

### Statistical analysis

The device oriented composite endpoint (DOCE) was defined as the primary outcome measure according to Academic Research Consortium (ARC) criteria [14]. Continuous variables were presented as mean ± one standard deviation and tested for significance with the t-test. The Chi-square test was used to compare categorical data in order to present them as counts and percentages. Kaplan-Meier estimates were calculated for DOCE, target lesion revascularization and scaffold thrombosis. In addition, to identify multivariable predictors for DOCE we performed a logistic regression analysis. Statistica release 7.1 Software (StatSoft Inc., Tulsa, OK, USA) was used to perform the calculation. Significance was supposed at a *p*-value of < 0.05.

## Results

There were 190 patients treated with Absorb BVS for lesions < 20 mm length and 136 patients treated with Absorb BVS for lesions longer or equal than 20 mm by quantitative coronary angiography.

Baseline characteristics were similar in both groups expect the number of diseased vessels as detailed in Table 2. The total lesion length was 11.6 ± 4.0 mm in the group with short lesions and 33.3 ± 14.0 mm in the group with long lesions. Consequently the length of the scaffolded segment was significantly longer in the group with long lesions compared to the group with short lesions. Maximal inflation pressure was significantly higher in long lesions. However, high-pressure post-dilatation with a non-compliant balloon was similar with 88% in short lesions and 89% in long lesions. Quantitative coronary analysis showed a significant higher acute gain in short lesions compared to long lesions, although post-procedural diameter stenosis did not differ (Table 3).

**Table 2:** Baseline Clinical Characteristics

	Lesion length < 20 mm	Lesion length ≥20 mm	*P*-values
Number of patients	190	136	
Age, years	61.4 ± 11.1	62.5 ± 9.8	0.35
Male sex, N (%)	152 (80.0)	100 (73.5)	0.17
Hypertension, N (%)	146 (76.8)	104 (76.5)	0.94
Diabetes mellitus, N (%)	44 (23.2)	23 (16.9)	0.17
Hyperlipidemia, N (%)	152 (80.0)	102 (75.0)	0.28
History of smoking, N (%)	98 (51.6)	76 (55.9)	0.33
Renal insufficiency, N (%)	16 (8.4)	16 (11.8)	0.61
Body mass index, kg/m^2^	27.8 ± 4.5	27.6 ± 4.9	0.64
Family history, N (%)	65 (34.2)	61 (44.9)	0.14
Number of diseased vessels	2.2 ± 0.8	2.5 ± 0.7	< 0.001
Stable angina, N (%)	106 (55.8)	77 (56.6)	0.88
ACS, N (%)	84 (44.2)	59 (43.4)	0.88

Data are presented as mean value±SD or percentage of patients. ACS: acute coronary syndrome

**Table 3:** Lesion Characteristics and Procedural Data

	Lesion length < 20 mm	Lesion length ≥20 mm	*P*-values
Number of Lesions	280	144	
Target vessel, N (%)			
LAD	138 (49.2)	62 (43.1)	0.002
CX	70 (25.0)	20 (13.9)	
RCA	69 (24.6)	61 (42.4)	
CABG	3 (1.1)	1 (0.7)	
AHA/ACC lesion type, N (%)			
A	21 (7.5)	0 (0.0)	< 0.001
B1	53 (18.9)	0 (0.0)	
B2	195 (69.6)	0 (0.0)	
C	11 (3.9)	144 (100.0)	
Bifurcation, N (%)	1 (0.4)	3 (2.4)	0.20
Lesion length, mm	11.6 ± 4.0	33.3 ± 14.0	< 0.001
Length of scaffolded segment, mm	22.6 ± 12.1	48.6 ± 23.6	< 0.001
High pressure post-dilation, N (%)	245 (87.5)	128 (88.9)	0.68
Maximal inflation pressure, atm	16.1 ± 3.0	17.1 ± 3.1	0.002
Reference diameter, mm (post)	2.94 ± 0.73	3.00 ± 1.28	0.57
Minimal lumen diameter, mm (post)	2.51 ± 0.44	2.51 ± 0.46	0.99
Diameter stenosis, % (post)	13.73 ± 8.15	13.34 ± 8.32	0.65
Acute gain	1.35 ± 0.48	1.06 ± 4.63	0.31

*LAD* left anterior descending artery, *CX* circumflex artery, *RCA* right coronary artery, *CABG* coronary artery bypass graft, *AHA* American Heart Association, *ACC* American College of Cardiology

The clinical follow-up after 12 months was completed in 98.5% of available patients. The rate of patients was 95.7% after 24 months and 90.8% after 36 months. The rate of scaffold thrombosis did not differ between groups and was 0% after 12 months, 1% after 24 months and 1.7% after 36 months in short lesions compared to 1.4, 0.0 and 0.0% in long lesions. Kaplan-Meier estimates for scaffold thrombosis after 12 months were 0% for short lesions and 1.40% for long lesions (*p* = 0.05), 0.46% vs. 1.40% after 24 months (*p* = 0.2286) and 4.05% vs. 1.50% after 36 months (*p* = 0.7529). After 12 months two definite scaffold thrombosis (after ARC-criteria) with total incidence of 0.48% and after 24 months one definite (total incidence: 0.30%) very late scaffold thrombosis occurred. Three possible (total incidence 1.68%) occurred in the 36 months follow-up.

**Fig. 1:**
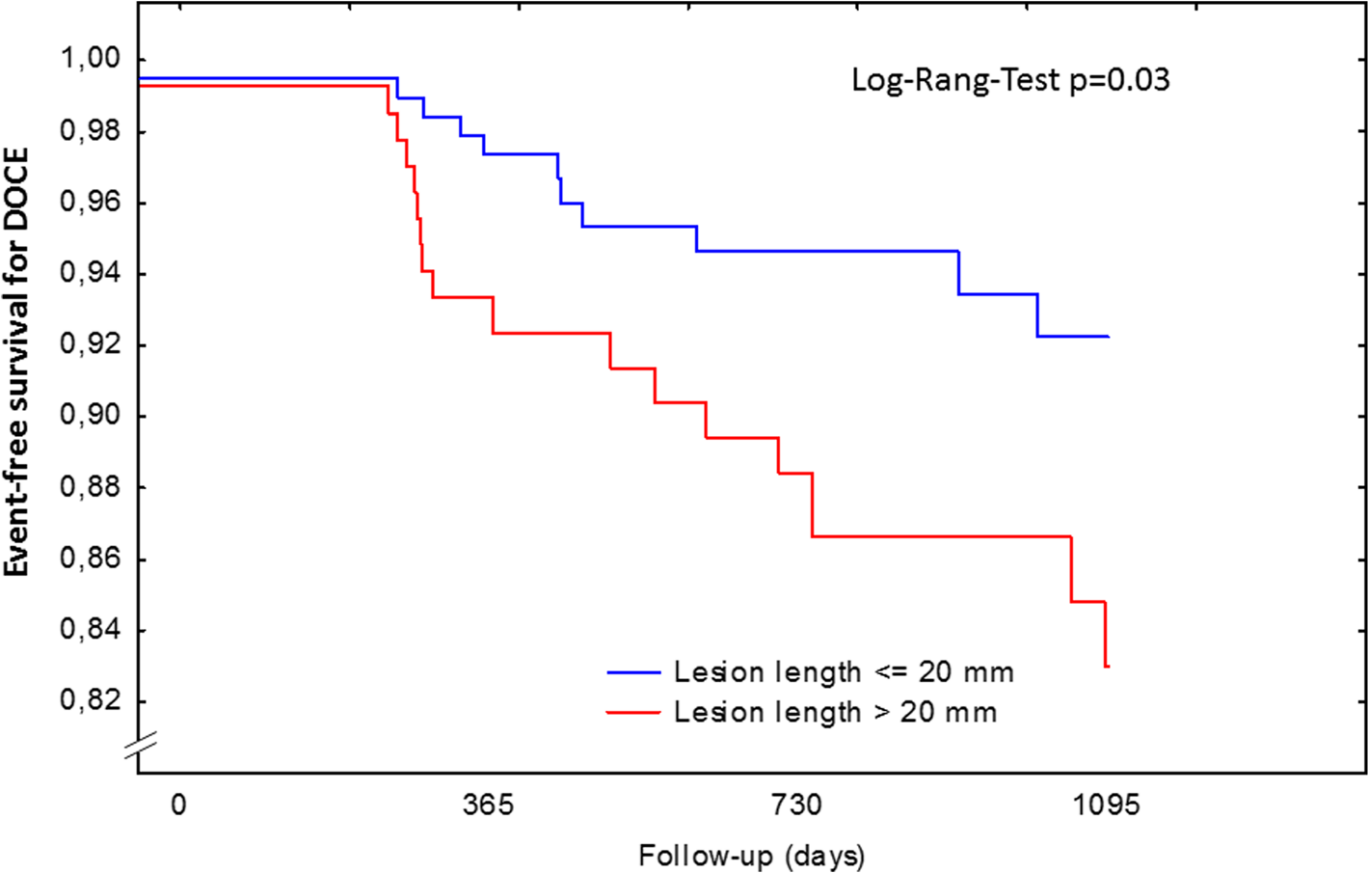
Kaplan-Meier analysis for event-free survival for DOCE (device oriented composite endpoint). DOCE was defined as cardiac death, myocardial infarction (MI) not clearly related to a non-target vessel and target lesion revascularization (TLR)

Target lesion revascularization was higher in long compared with short lesions after 12, 24 and 36 months (Tables 4, 5 and 6). Kaplan-Meier estimates for target lesion revascularization after 12 months were 1.46% for short and 7,69% for long lesions (*p* = 0.0012), 2.06% vs. 8.75% after 24 months (*p* = 0.0027) and 4.96% vs. 9.59% after 36 months of follow-up (*p* = 0.0109). Consequently the DOCE was numerically higher in patients with long lesions as compared to patients with short lesions. The difference between groups was 6.2, 4.4 and 5.1% after 12, 24 and 36 months favoring shorter lesions. Kaplan-Meier estimates for DOCE after 12 months were 2.63% for short lesions and 8.09% for long lesions (*p* = 0.0131), 5.51% vs. 11.35% (*p* = 0.0503) after 24 months and 8.00% vs. 18.00% (*p* = 0.0264) after 36 months of clinical follow-up (Fig. 1).

**Table 4:** Device-oriented endpoint within 12 months

	Total	Lesion length < 20 mm	Lesion length ≥20 mm	*P*-Value
Number of lesions	417	274	143	
Scaffold thrombosis, N (%)				
acute	0 (0)	0 (0)	0 (0)	0.04
subacute	1 (0.2)	0 (0.0)	1 (0.7)	
Late	1 (0.2)	0 (0.0)	1 (0.7)	
TLR, N (%)	14 (3.4)	4 (1.5)	10 (7.0)	0.01
MI, N (%)	6 (1.4)	2 (0.7)	4 (2.8)	0.14
Number of patients	321	186	135	
DoCE, N (%)	17 (5.3)	5 (2.7)	12 (8.9)	0.015
Cardiac mortality, N (%)	2 (0.6)	0 (0.0)	2 (1.5)	0.10

*TLR* ischemia-driven target lesion revascularization, *MI* myocardial infarction, not clearly related to a non-target vessel, *DoCE* device-oriented endpoints

**Table 5:** Device-oriented endpoint within 24 months

	Total	Lesion length < 20 mm	Lesion length ≥20 mm	*P*-Value
Number of lesions	329	223	106	
Scaffold thrombosis, N (%)				
	1 (0.3)	1 (0.4)	0 (0.0)	0.38
TLR, N (%)	14 (4.3)	5 (2.3)	9 (8.7)	0.03
MI, N (%)	7 (2.1)	4 (1.8)	3 (2.8)	0.15
Number of patients	247	146	101	
DoCE, N (%)	18 (7.3)	8 (5.5)	10 (9.9)	0.19
Cardiac mortality, N (%)	3 (1.2)	2 (1.4)	1 (1.0)	0.96

*TLR* ischemia-driven target lesion revascularization, *MI* myocardial infarction, not clearly related to a non-target vessel; *DoCE* device-oriented endpoints

**Table 6:** Device-oriented endpoint within 36 months

	Total	Lesion length < 20 mm	Lesion length ≥ 20 mm	*P*-Value
Number of lesions	179	121	58	
Scaffold thrombosis, N (%)	3 (1.1)	3 (1.7)	0 (0.0)	0.12
TLR, N (%)	11 (6.1)	5 (4.1)	6 (10.3)	0.08
MI, N (%)	8 (4.5)	5 (4.1)	3 (5.2)	0.21
Number of patients	139	84	55	
DoCE, N (%)	16 (11.5)	8 (9.5)	8 (14.6)	0.37
Cardiac mortality, N (%)	5 (3.6)	3 (3.6)	2 (3.6)	0.95

*TLR* ischemia-driven target lesion revascularization, *MI* myocardial infarction, not clearly related to a non-target vessel; *DoCE* device-oriented endpoints

To get a better idea of the population reaching an endpoint during the follow-up period, we divided the population up into one group without events and one group with events. Baseline clinical characteristics were similar in both groups expect renal insufficiency and family history. There were significant more patients suffering from renal insufficiency in the event group (28.6% vs. 8.1%, *p* = 0.009; 2a). Significant more patients with positive family history concerning cardiovascular events were in the group without events over the follow-up period (38.9% vs. 35.7%, *p* = 0.02 Table 7).

**Table 7:** Baseline Clinical Characteristics

	Patients without Event	Patients with Event	*P*-values
Number of patients	298	28	
Age, years	61.7 ± 10.6	64.1 ± 9.5	0.24
Male sex, N (%)	231 (77.5)	21 (75.0)	0.76
Hypertension, N (%)	226 (75.8)	24 (85.7)	0.21
Diabetes mellitus, N (%)	59 (19.8)	8 (28.6)	0.29
Hyperlipidemia, N (%)	234 (78.5)	20 (71.4)	0.40
History of smoking, N (%)	154 (51.7)	20 (71.4)	0.22
Renal insufficiency, N (%)	24 (8.1)	8 (28.6)	0.009
Body mass index, kg/m^2^	27.7 ± 4.7	27.4 ± 5.0	0.74
Family history, N (%)	116 (38.9)	10 (35.7)	0.02
Number of diseased vessels	2.3 ± 0.8	2.4 ± 0.7	0.38
Stable angina, N (%)	167 (56.0)	16 (57.1)	0.91
ACS, N (%)	131 (44.0)	12 (42.9)	0.91

Data are presented as mean value±SD or percentage of patients. ACS: acute coronary syndrome

As expected the lesions in the event-group were significant longer (17.8 ± 12.7 vs. 24.3 ± 16.6; *p* = 0.01) and diameter stenosis (post) was significant higher (16.75 ± 9.05 vs. 13.37 ± 8.09, *p* = 0.04 see Table 8). In addition, we performed a logistic regression analysis to identify predictors for device failure (DoCE). We included diabetes mellitus, renal insufficiency, lesion in left anterior descending, history of smoking, acute coronary syndrome and lesion length (short versus long lesions). The following variables were significant predictors for device failure: renal insufficiency (OR 4.60 95% CI 1.95–10.9, *p* < 0.001), lesion in left anterior descending (OR 3.52 95% CI 1.47–8.42, *p* < 0.01) and long lesions (OR 2.50 95% CI 1.13–5.57, *p* = 0.024) whereas presence of diabetes mellitus (*p* = 0.14), history of smoking (*p* = 0.07), acute coronary syndrome (*p* = 0.44) were not predictive.

**Table 8:** Lesion Characteristics and Procedural Data

	Without Event	With Event	*P*-values
Number of Lesions	394	30	
Target vessel, N (%)			
LAD	178 (45.3)	22 (73.3)	0.009
CX	84 (21.3)	6 (20.0)	
RCA	128 (32.5)	2 (6.7)	
CABG	4 (1.0)	0 (0.0)	
AHA/ACC lesion type, N (%)			
A	21 (5.3)	0 (0.0)	0.06
B1	50 (12.7)	3 (10.0)	
B2	185 (47.0)	10 (33.3)	
C	138 (35.0)	17 (56.7)	
Bifurcation, N (%)	4 (1.1)	0 (0.0)	0.64
Lesion length, mm	17.8 ± 12.7	24.3 ± 16.6	0.01
Length of scaffolded segment, mm	30.9 ± 20.5	38.8 ± 25.1	0.05
High pressure post-dilation, N (%)	347 (88.1)	26 (86.7)	0.82
Maximal inflation pressure, atm	16.4 ± 3.0	16.5 ± 3.5	0.92
Reference diameter, mm (post)	2.97 ± 0.97	2.86 ± 0.35	0.53
Minimal lumen diameter, mm (post)	2.52 ± 0.45	2.38 ± 0.41	0.99
Diameter stenosis, % (post)	13.37 ± 8.09	16.75 ± 9.05	0.04
Acute gain	1.26 ± 2.69	1.31 ± 0.36	0.31

*LAD* left anterior descending artery, *CX* circumflex artery, *RCA* right coronary artery, *CABG* coronary artery bypass graft, *AHA* American Heart Association, *ACC* American College of Cardiology

## Discussion

Using PSP-technique in 88% of lesions treated with Absorb BVS we were able to demonstrate that long lesions have a higher rate of TLR but not a higher risk for scaffold thrombosis up to 36 months of follow-up.

Kaplan-Meier estimates showed significant differences concerning DOCE after 12, 24 and 36 months. Kaplan-Meier-estimates for target lesion revascularization demonstrated significant lower rates in short lesions. For scaffold thrombosis no significant difference between short and long lesions concerning Kaplan-Meier estimates were observed.

Former studies especially the ABSORB-Trials showed good clinical results in selected lesions after 12 months follow-up [2, 15–17]. Regarding scaffold thrombosis rates an alarmingly trend towards higher rates of thrombotic events in patients treated with bioresorbable scaffolds could be seen. Considering scaffold thrombosis the randomized ABSORB-II trial with a follow-up period of three years showed a high scaffold thrombosis rate of 3% compared to 0% in metallic drug-eluting stents [1]. Causal for these findings missing predilation, wrong size selection and the lack of careful postdilation can be mentioned. Proper studies confirmed these findings in metallic drug eluting stents. Because of the strut thickness a careful implantation technique with predilation, proper sizing and postdilation is essential. As it is known from the one year analysis of ABSORB II the postdilation in this study was 61% [15].

The Absorb III study enrolled 2008 patients and randomized them 2:1 for treatment with scaffolds or drug-eluting stents [2]. After one year the scaffold thrombosis rate including probable or definite scaffold thrombosis was 1.5% in the bioresorbable scaffold group and 0.7% in the metallic drug-eluting-stent population without reaching significance [2]. Postdilation after scaffold implantation in this study was 65.5% [2]. The three-year data of the ABSORB-Trial in low and moderate complex lesions was associated with low and acceptable rates of major adverse clinical events with the proper PSP-technique. The ABSORB-Investigators could also demonstrate that the scaffold thrombosis rate was higher than with metallic DES [18].

The AIDA study enrolled 1845 patients and randomized them either in the bioresorbable scaffold group or the drug-eluting stent group [3]. They found no significant difference between the treatment with bioresorbable scaffolds or with metallic drug-eluting stents concerning target-vessel failure in a two year period (11.7% vs. 10.7%, *p* = 0.43) [3]. After 2 years of follow-up a higher rate of device thrombosis could be observed (3.5% vs. 0.9%, p = < 0.001) [3]. In this study predilation took place in 96.9% of treated patients and postdilation was used in 74% of patients [3].

In the GHOST-EU registry patients with exclusion criteria for randomized ABSORB trials were enrolled to represent a real-world population [19]. The scaffold thrombosis rate over 6 months was 2.1% [19]. A real-world population was studied by GABI-R as well. The GABI-R Investigators divided the population up into patients with scaffold thrombosis and patients without this thrombotic event to study predictors. In this international multicenter study 3137 patients were included between November 2013 and January 2016. Predilation took place in 95.6% of cases and postdilation was present in 77.3% of lesions. Differences between these two populations could be seen in a longer median scaffolded length (28 mm vs. 23 mm), a higher rate of scaffolded bifurcations (11.8% vs. 3.7%, *p* = < 0.0001) and more ostial lesions (3.9% vs. 0.8%, p = < 0.05).

Analyzing the patients with scaffold thrombosis, a longer scaffold length of 20.45 ± 5.66 mm (without scaffold thrombosis: 19.65 ± 6.23 mm; *p* = 0.27) and lower rates of postdilation (65.7% vs. 73.3%) were present [Differences between patients with and patients without scaffold thrombosis – Results of the German-Austrian ABSORB RegIstRy (GABI-R), J. Wöhrle and GABI-R Investigators, DGK 2017].

The idea that bioresorbable scaffolds could prove their benefits by regained vasomotion and complete resorption of the vessel-cage could only be studied at very long follow-up periods [19]. Therefore we planned to continue the follow-up time of this study until 10 years.

The 36 months data from the ABSORB II trial demonstrated a significant higher rate of adverse events including thrombotic ones using the bioresorbable vascular scaffold instead of metallic drug-eluting stents [1]. On top the study failed to reach the endpoint in late lumen loss [20]. In this study the PSP-technique was not routinely used. Meta-analysis summarizing important randomized studies showed a higher rate of scaffold thrombosis comparing to common metallic drug eluting stents [21–23]. Astonishingly the postdilation rate was between 52 and 66% [21–23]. Fernandes et al. analyzed a real world population with long coronary lesions after implantation of an everolimus-eluting stent [7]. The MACE and ST rates at 12, and 24-months follow-up were 2.1, 5.4% (MACE) and 0.7, 1.5% (ST) [7]. The scaffold thrombosis rate in our study was 1.4, 0.0% and for DOCE 8.9, 9.9%, after 12 and 24-month follow-up. Lesiak et al. compared a bioresorbable polymer sirolimus-eluting stent in patients with long coronary lesions with permanent polymer everolimus-eluting stent [8]. The target lesion revascularisation rate was 3.7% and the scaffold thrombosis rate was 1.2% in EES after 9 months of follow-up [8]. A recent study by Kang et al. demonstrated good results in very long coronary lesions (> 50 mm) with zotarolimus- and everolimus drug eluting stents [9]. Patra et al. showed 4% TLF after 12 months of follow up [10]. It is known that the bioresorbable scaffold has higher DOCE rates in a real world population. This comparison with results of the literature demonstrate that Absorb BVS is associated with a higher event rate compared with everolimus eluting stents [24].

With the introduction of the implantation-technique of Puricel and Gori et al. a significant reduction of scaffold thrombosis could be seen [4]. This implantation technique is composed of predilation with a non-compliant balloon up to the reference vessel diameter, then implantation of the scaffold of same size and careful high-pressure postdilation with a non-compliant balloon 0.5 mm larger than the implanted device – the nowadays so called PSP-technique [4]. It is to be assumed that careful and accurate implantation technique as well as the learning curve of interventionalists – as seen in GABI-R have a great impact on our outcome measures and especially thrombotic events [25]. The idea that bioresorbable scaffolds could prove their benefits by regained vasomotion and complete resorption of the vessel-cage could only be studied at very long follow-up periods [20]. Tanaka et al. showed a low scaffold thrombosis rate (1.2%) after a two year follow-up using the bioresorbable vascular scaffold in a population of 264 patients with 400 lesions with careful PSP-technique [26].

Sotomi et al. studied in his OCT-controlled population the most common reasons for scaffold thrombosis [27]. Malapposition (24%), incomplete lesion coverage (18%) and device underexpansion (12%) were reasons for early scaffold thrombosis [27]. Late ones fail at malapposition (35%), discontinuity (31%), peri-strut low intensity area (19%) and incomplete lesion coverage (12%) [27]. Also the device overlap seems to be a factor increasing the scaffold thrombosis rate [28]. The study group of Polimeni et al. enrolled 183 patients with ST-segment myocardial infarction in their study and could demonstrate that the scaffold thrombosis rate was reduced when an optimized implantation technique was used [29]. Many recent studies tried to find predictors for scaffold failure and figured out that the implantation technique is a very important factor in reducing cardiac events [28, 30–36]. The use in acute coronary syndroms studied by Anadol et al. seems to be safe as well [37]. But for long coronary lesions.

In our study after 12 months there was a significant difference concerning scaffold thrombosis between the both different lesion length groups (long lesion length 0.7% vs. 0% in short lesions). With the use of PSP-technique we could show low scaffold thrombosis rates and low target lesion revascularizations. In the UNDERDOGs study analyzing long coronary lesions requiring overlap the scaffold thrombosis rate in total after the whole follow-up period of up to 24 month was 1.2% [38]. The target lesion revascularization rate was identical in the UNDERDOGs study compared with our study (4.3%). After 24 months a significant higher rate of target lesion revascularizations could be seen in the longer lesion group (8.7% vs. 2.3%) underlining the purpose that longer lesions bear a higher risk for reinterventions. However using the PSP-technique in a respective moderate lesion number no higher scaffold thrombosis rate could be seen. Wiebe et al. studied the scaffold implantation in long lesions (minimum 28 mm) with overlapping scaffolds. They enrolled 250 patients and at 12-months follow-up a scaffold thrombosis rate of 2,3% could be seen (0.7% in our study) [39]. The authors Geraci et al. investigated in a subgroup analysis from the GHOST-EU registry 1-year outcomes in patients with long coronary lesions treated with bioresorbable everolimus-eluting scaffolds. The lesions were divided into three groups: < 30 mm; 30-60 mm; ≥60 mm scaffolded length. As assumed they could demonstrate that patients with longer lesions had more comorbidities and more complex lesion characteristics like chronic total occlusions and bifurcation lesions [27]. Compared to 30-60 mm lesion length group the scaffold thrombosis rate was 1.1% vs. 0.7% in our study [40]. They demonstrated that patients with long coronary lesions (over 60 mm) treated with bioresorbable scaffolds had higher TLF rates, driven by myocardial infarction and clinically driven target lesion revascularization [40] (Table 9).

**Table 9:** Scaffold thrombosis rates and target lesion revascularisation after 12 months

Authors	*n*	Lesion length mm	Scaffold thrombosis	TLR
UNDERDOGs [38]	162	54 ± 15	1.2%	4.3%
Geraci et al. [40]	276	30–60	1.1%	4.5%
Wiebe et al. [39]	250	≥28	2.3%	4.0%
Present data	143	≥20	1.4%	7%

In conclusion the implantation of bioresorbable vascular scaffolds Absorb in long lesions compared to short ones is safe using the PSP-technique including accurate predilation, proper sizing and postdilation and shows an acceptable risk of thrombotic or adverse events.

### Limitations

This prospective study was non-randomized and could therefore contain selection bias. Another limitation is the small sample size therefore larger randomized studies are necessary to prove our findings. In well-known randomized studies the thrombosis rates are higher than in our study although we carefully used the PSP technique.

## Conclusion

In long lesions compared to short ones the bioresorbable scaffold Absorb has higher DoCE-rates but shows acceptable clinical follow-up results up to 36 months with the proper PSP-technique.
